# Ileal Microbiota Alters the Immunity Statues to Affect Body Weight in Muscovy Ducks

**DOI:** 10.3389/fimmu.2022.844102

**Published:** 2022-02-10

**Authors:** Zixian Fu, Hua Yang, Yingping Xiao, Xiaoli Wang, Caimei Yang, Lizhi Lu, Wen Wang, Wentao Lyu

**Affiliations:** ^1^ State Key Laboratory for Managing Biotic and Chemical Threats to the Quality and Safety of Agro-Products, Institute of Agro-product Safety and Nutrition, Zhejiang Academy of Agricultural Sciences, Hangzhou, China; ^2^ College of Animal Science, Zhejiang A&F University, Hangzhou, China; ^3^ Institute of Animal Husbandry and Veterinary Science, Zhejiang Academy of Agricultural Sciences, Hangzhou, China

**Keywords:** body weight, ileal microbiota, Muscovy ducks, microbial composition, growth-related bacteria, immunity

## Abstract

The ileum is mainly responsible for food absorption and nutrients transportation. The microbes in its intestinal lumen play an essential role in the growth and health of the host. However, it is still unknown how the ileal microbes affect the body weight of the host. In this study, we used Muscovy ducks as an animal model to investigate the relationship between the ileal microbes and body weight and further explore the potential mechanism. The ileum tissue and ileal contents of 200 Muscovy ducks were collected for mRNA extraction and real-time quantitative PCR, as well as DNA separation and 16S rRNA gene sequencing. With body weight being ranked, the bottom 20% (n = 40) and top 20% (n = 40) were set as the low and high groups, respectively. Our results showed that in the ileum of Muscovy ducks, the Bacteroides, Firmicutes, and Proteobacteria were the predominant phyla with the 10 most abundant genera, namely *Candidatus Arthromitus*, *Bacteroides*, *Streptococcus*, *Vibrio*, *Romboutsia*, *Cetobacterium*, *Clostridium sensu stricto 1*, *Terrisporobacter*, *Escherichia-Shigella*, and *Lactobacillus.* We identified *Streptococcus*, *Escherichia-Shigella*, *Candidatus Arthromitus*, *Bacteroides*, *Faecalibacterium*, and *Oscillospira* were closely correlated to the growth of Muscovy ducks. *Streptococcus* and *Escherichia-Shigella* were negatively related to body weight (BW), while *Candidatus Arthromitus*, *Bacteroides*, *Faecalibacterium*, and *Oscillospira* were positively associated with BW. In addition, we found that the relative expression levels of tight junction proteins (*Claudin 1*, *Claudin 2*, *ZO-1* and *ZO-2*) in the high group showed an upward trend, although this trend was not significant (*P* > 0.05). The expression of pro-inflammatory factors (*IL-1β*, *IL-2* and *TNF-α*) decreased in the high group, while the anti-inflammatory factor *IL-10* increased. Of course, except *IL-2*, these differences were not significant (*P* > 0.05). Finally, the correlation analysis showed that *Escherichia-Shigella* was significantly positively correlated with *IL-1β* (*P* < 0.05). These findings may provide fundamental data for the development of next-generation probiotics and assist the development of strategies for changing the gut microbiota to promote the growth performance in the duck industry.

## Introduction

It is widely predicted that the world’s population will increase to nine billion by 2050, resulting in an increased require milk, meat, and other animal products (1). Poultry meat will become the most consumed animal meat globally (2). In the past 50 years, animal genetics and animal nutrition have made tremendous progress in meeting the increasing demand, particularly in poultry. As a kind of common farm animals in the world, ducks are a powerful experimental model for basic and applied research. It is commonly used in duck-derived disease research (3) and virus research for human (4, 5). Muscovy ducks (Cairina moschata) have been domesticated for hundreds of years after being introduced into China. They are trendy among consumers because of their strong-tasting meat with tenderness and leanness (6). Furthermore, Muscovy ducks are high-quality meat ducks, which not only have a high lean meat rate and low subcutaneous fat content but are also rich in nutritional value. The meat from Muscovy ducks contains various amino acids, unsaturated fatty acids, exhibiting high content of vitamin B, vitamin E, and trace elements such as zinc, copper, iron, etc (7). Therefore, Muscovy ducks have a high commercial value in addition to the general characteristics of ducks.

The intestine is an organ responsible for nutrient digestion and absorption and plays a crucial role in immune response, microorganism defense and hormone secretion (8). Trillions of microbes inhabit the intestines of humans and complex metazoans. Their function is to harvest, store and expend the energy obtained from diets for the host as an additional organ (9). Due to participating in the development of the intestinal epithelium and the regulation of physiological functions to maintain the homeostasis of nutrients, digestion, intestinal barrier function, and immunity, the structure and functionality of the gut microbiota are indispensable to the health of thehost (10). Immunity is one of the essential factors affecting host body weight. Many studies have proved that the intestinal microbial disorder can cause the host’s pro-inflammatory response, leading to various intestinal diseases, and the final intuitive manifestation is the host’s weight loss (11, 12). Therefore, the intervention of intestinal microbes might help to optimize the structure of the host flora, enhance the body’s immunity, increase the efficiency of food absorption and energy consumption, and thereby promote weight (13). In fact, many studies have proved that the addition of probiotics in diets can improve the structure of intestinal microbes, enhance immunity and increase the growth performance of the host (14–16). Alternatively, early colonization of probiotics (17, 18) and fecal microbiota transplantation (19) can accelerate the maturation of the intestinal microbes and improve the growth performance of the host. To date, most investigations on the ileal microbiota in poultry is about chickens (20–23). However, little is known about the bacteria in the ileum of Muscovy ducks. To identify the specific bacteria, which might potentially regulate the growth performance and immunity. In the present study, we detected the bacterial composition of the ileum of 200 Muscovy ducks, and compared the ileal microbiota and immune-related factors between the ducks with the highest and lowest body weight (BW). This study would provide basic data to promote the development of next-generation probiotics, and supply insights into the ileal microbial community and the association of ileal microbiota with the growth performance in Muscovy ducks.

## Materials and Methods

### Compliance With Ethical Standards

The experiments were authorized by the Animal Care and Use Committee of the Zhejiang Academy of Agricultural Sciences (2019ZAASLA37).

### Ducks and Sample Collection

A population of newly-hatched 5000 Muscovy (*C. moschata*) ducks (Lanxi Hewang Breeding Duck Co., Ltd., Jinhua, China) were fed with a commercial diet and water *ad libitum* under standardized conditions for 70 days. The diet composition was as previously described (24). Two hundred ducks were randomly selected from a 5000 population. After being weighed, ducks were euthanized by cervical dislocation. The ileal segment and content of each duck were gathered immediately, frozen in liquid nitrogen, and stored at -80°C until the mRNA and DNA were separated.

### DNA Isolation and Sequencing

Host genomic DNA was separated from each ileum sample using a QIAamp DNA Stool Mini Kit (Qiagen, Valencia, CA) following the manufacturers’ instructions. Use 1% agarose gel electrophoresis and NanoDrop ND-1000 (Thermo Fisher Scientific, Waltham, MA, USA) to evaluate the quality and concentration of the DNA extract. Use next-generation sequencing to sequence high-quality DNA (25). In detail, the V4-V5 region of the bacterial 16S rRNA gene was amplified by the barcode-fusion forward primer 515F (5’-GTGCCAGCMGCCGCGGTAA-3’) and the reverse primer 907R (5’- CCGTCAATTCMTTTRAGTTT -3’). The conditions of PCR were as previously described (26). After PCR, the 2% (w/v) agarose gel was used to separate and qualify the amplicons and then purified using a GeneJET Gel Extraction Kit (Thermo Scientific). An Illumina TruSeq DNA PCR-Free Library Preparation Kit (Illumina) was appropriated for sequencing library generation. An Agilent Bioanalyzer 2100 System and a Qubit 2.0 Fluorometer (Thermo Scientific) were used to evaluate the quality of the generated library. The qualified library was sequenced commercially using Mingke Biotechnology (Hangzhou) on an Illumina NovaSeq platform, producing a 250 bp paired-end read.

### Bioinformatics Analysis of Ileum

The Illumina paired-end reads were demultiplexed and filtered in Quantitative Insights into Microbial Ecology (QIIME) quality filters (27) to remove low-quality reads, merged into tags by FLASH (28), and assorted each sample according to a unique barcode. Analyze the label of each sample after removing redundancy, and the UPARSE and UCHIME were used to assign unique tags with ≥ 97% sequence similarity to the same operational taxonomic units (OTUs).

The RDP classifier was used to annotate the classification information of the selected OUT (29). Alpha-diversity (Observed Species, Chao 1 estimator, ACE, Shannon, and Simpson indices) was calculated and visualized using GraphPad Prism 8 (GraphPad Software, San Diego, CA, USA). The rooted phylogenetic tree was visualized in the Interactive Tree of Life (iTOL, https://itol.embl.de/; version 5.5).

### Real-Time Quantitative PCR (RT-qPCR)

Total RNA in the ileal tissue was extracted by Trizol^®^ Plus RNA Purification Kit (Thermo Fisher) in accordance with the manufacturer’s instructions. Briefly, 50-100 mg tissue samples were ground into a powder with a tissue homogenizer, and then transferred to a tube with 1 ml of Trizol and incubated at room temperature for 5 mins. Added 0.2 ml chloroform and shook the tube vigorously by hand for 15 s, then incubated at room temperature for 2-3 mins. After being centrifuged at 12000 × g for 15 mins at 4°C, the supernatant was transferred to a new tube, then RNA was precipitated with an equal volume of 70% ethanol. The quantity and quality of RNA were evaluated by a spectrophotometer (NanoDrop-2000, Thermo Fisher Scientific, MA, USA). cDNA was synthesized by SuperScript™ III First-Strand Synthesis SuperMix (Thermo Fisher). RT-qPCR was performed on an ABI prism 7700 Sequence Detector System (Applied Biosystems, Foster City, CA, USA). The reaction scheme was as follows: pre-incubation at 95°C for 1 min, then performing 40 cycles of denaturation at 95°C for 15 s and annealing at 60°C for 25 s. Then, a melting curve analysis was conducted to affirm the specificity and reliability of the PCR products. Using glyceraldehyde 3-phosphate dehydrogenase (GAPDH) as the internal reference gene, the relative expression of mRNA was calculated by the2^−ΔΔCt^ method (30). The primers designed in this study are listed in **Table 1**.

**Table 1:** Primers for gene expression analysis using RT-Qpcr.

Gene	GenBank accession	Primer Sequence (5’-3’)	Expected size (bp)	
*ZO-1*	XM_027465580.1	F: GAAGAGGTGGCAGGCGAAA R: GACTGACTGGTAAATCCACATC	107	
*ZO-2*	XM_027446904.1	F: GAGCAGGGGAAGGAGCAT R: CTCACCTCCCTCACGTATCT	120	
*Claudin1*	XM_013108556.3	F: CCGTGACTGGCATGAAATGCA R: CACCAATGCTGACAAACCTGCAA	113	
*Claudin2*	XM_021271062.2	F: CCTACATCGGCTCCAGCAT R: GGTTGAGCATGGAGCTGTAGAT	117	
*IL-1β*	XM_038166869.1	F: TCATCTTCTACCGCCTGGACG R: TAGGTGGCGATGTTGACCT	148	
*IL-2*	NM_001310373.1	F: CCAGGAACGGGATGCAATATCTGTG R: CTCAGGAAGTTGGTCAGCTCTTGG		80
*IL-10*	NM_001310368.1	F: CAACCTGCTGCTGAGCCTGAAG R: CGCCTTGTAGATGCCGTTCTCG	133	
*TNF-α*	XM_005027491.5	F: ACAGGACAGCCTATGCCAAC R: ACAGGAAGGGCAACACATCT	111	
*GAPDH*	XM_005016745.2	F: GGAGCTGCCCAGAACATTATC R: GCAGGTCAGGTCCACGACA	141	

### Identify the Relationship Between Growth-Related Microbiota and Immunity

To determine the relationship between growth-related microbiota and immunity. First, we precluded the taxa present in <30% of Muscovy duck ileum samples to detect which microorganisms were significantly related to BW. All the samples were then successively ranked by host BW and the relative abundance of each microorganism. The highest 20% and lowest 20% of the sorted ducks were regarded as two different groups, and all traits between the two groups were statistically analyzed. Subsequently, in the characterization of body weight-related bacteria (detected in at least 30% of ileum samples), static analysis of the BW between the highest 20% and the lowest 20% relative abundance of common bacteria was performed. Finally, the growth-related microbiota and immune-related values were used for correlation heatmap analysis to explore the relationship between them.

### Co-Occurrence Network and Correlation Heatmap Analysis

The correlation network analysis was performed based on Spearman’s correlation matrixes among the relative abundance of each genus. Networks were visualized by using Gephi v0.9.2 software (France) (31). The correlation heatmap analysis was performed based on the Pearson algorithm among the relative abundance of each genus and immunity value.

### Data Analysis

Data are expressed as the mean ± SD. All data analyses were visualized using GraphPad Prism 8 software. Unpaired two-tailed Student’s t-test analyzed the difference between the two groups.t. When the *P*-value did not exceed 0.05, it was considered significant.

### Accession Number

The original sequencing reads of this study have been deposited in NCBI with the deposit number of BioProject PRJNA762153.

## Results

### Microbiota Composition in the Ileum of Muscovy Ducks

A total of 8,360,552 high-quality reads were generated with an average of 41,803 reads in each sample, and were classified into 7035 bacterial OTUs based on 97% sequence similarity. Then these OTUs were assigned into 49 phyla, 136 classes, 332 orders, 572 families, 1323 genus, and 1989 species. We first characterized the bacterial composition in the ileum of Muscovy ducks that may be related to growth.

**Figure 1:**
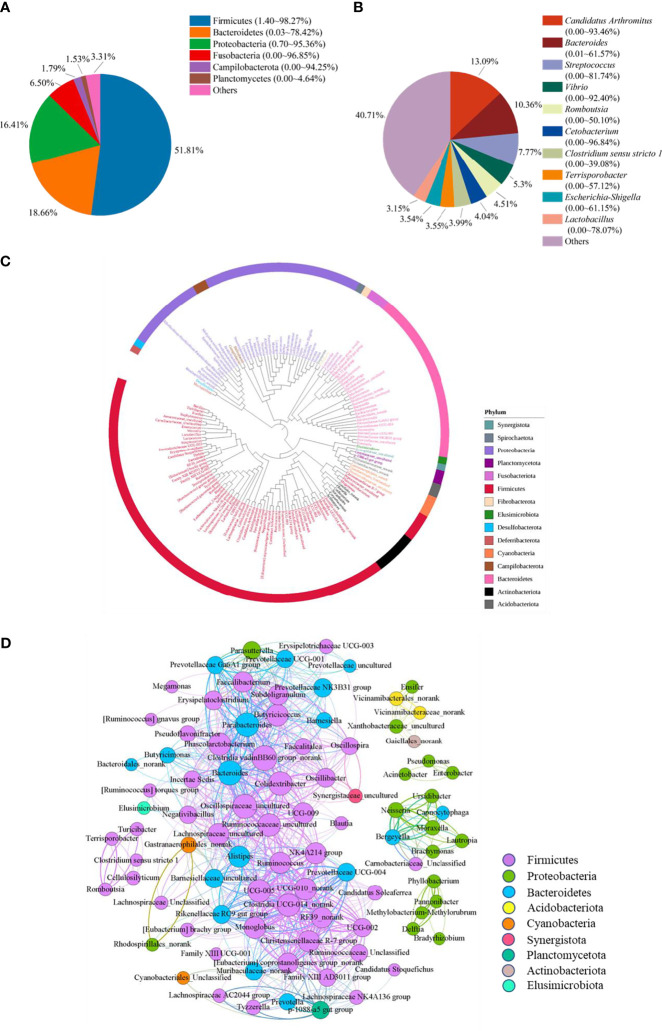
The microbiota composition in the ileum of Muscovy ducks. Two hundred newly hatched ducklings were fed commercial feeds for 70 days before the ileal content samples were collected from each ileum segment. The relative abundance of OTUs was used to determine the bacterial composition at phylum **(A)** and genus **(B)** levels with 200 samples. Only the top six phyla and 10 genera are displayed, with unidentified and lowly abundant bacteria collectively denoted as “others”. The phylogenetic tree **(C)** and co-occurrence pattern **(D)** of 50% the common bacteria in the ileum of Muscovy ducks. In the phylogenetic tree, the colored strips and labels were colored by the phylum. In the co-occurrence network, the sold line indicated that Spearman’s rank correlation coefficient > 0.8 with *P* < 0.001; the size of the node was proportional to the abundance of the genus; the color of the nodes and lines were related to the phylum to which the genus belongs.

To reveal the bacterial composition in the ileum of Muscovy ducks, the relative abundance was calculated at both the phylum and genus levels. The top six dominant phyla were Firmicutes, Bacteroidetes, Proteobacteria, Fusobacteria, Campilobacterota, and Planctomycetes, representing 69.84% to 99.95% of the total bacterial population, respectively. Among these phyla, Firmicutes accounted for 1.40% to 98.27% of the bacteria while Bacteroidetes amounted to 0.03% to 78.42% (**Figure 1A**). Planctomycetes was the minor phylum detected at an average of 1.53% in the ileum. Additionally, the minor phyla and unidentified bacteria were denoted as “Others”.

At genus level, ten most abundant genera were Candidatus Arthromitus, Bacteroides, Streptococcus, Vibrio, Romboutsia, Cetobacterium, Clostridium sensu stricto 1, Terrisporobacter, Escherichia-Shigella, and Lactobacillus (**Figure 1B**). Obviously, the ileal microbiota appeared to be very diverse, with the top 10 genera accounting for an average of 59.29%, ranging from 1.94% to 98.11%, in relative bacterial abundance in the ileum. Additionally, these genera had a similar relative abundance. The relative abundance of the top three genera was around 10%, while the abundance of the following seven genera was around 5%.

Next, to reveal the interaction among the common genera in the ileum of Muscovy ducks, we calculated the number of common bacteria genera presenting in 50% of the ileum samples, which accounted for 11.48% of all the genera in the ileum, and generated the phylogenetic tree and the co-occurrence network based on the Spearman’s correlation among the representative bacteria in the ileum. The result showed that among the phyla categorized from the 149 common genera, Firmicutes, Bacteroidetes and Proteobacteria were the top three phyla (**Figures 1C, D**). Meanwhile, among these positively related bacterial genera, most Firmicutes, Bacteroidetes and Proteobacteria formed an extensive co-occurrence network with other bacterial genera, and a few formed several independent, small and stable clusters. Some phyla with a small number of genera, such as *Acidobacteria* and *Actinobacteria*, were only associated with a few genera to form a small co-occurrence network. In contrast, *Cyanobacteria* and *Planctomycetata* were merely part of an extensive co-occurrence network and were related to multiple genera (**Figure 1D**). These results revealed that the relationship between ileal bacteria was diverse and complex.

### The Growth Performance of Muscovy Ducks Differed Between the High Group and the Low Group

**Figure 2:**
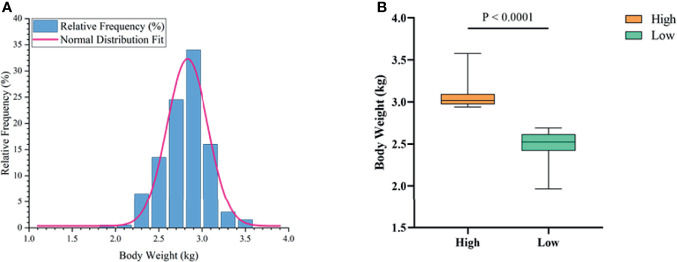
The phenotype of Muscovy Ducks. Two hundred newly hatched ducklings were fed commercial feeds for 70 days before being weighed individually and collected the ileal segment and contents after dissection. The highest 20% and the lowest 20% of the BW-ranked ducks were set as the high and low groups from the 200 Muscovy ducks, respectively. The test of a normal distribution on the BW **(A)** and the phenotype of BW **(B)** in high and low groups. Data were expressed as mean ± SD (n = 40) and analyzed by unpaired two-tailed Students’ t-test.

To determine the growth performance of Muscovy ducks, we weighed the ducks individually and collected ileal segment and content after dissection. We first tested whether the BW data fit a normal distribution before further data analysis. As expected, the BW gave a classical inverted-bell curve (Gauss Fit, *P* < 0.0001, **Figure 2A**). Next, we set the highest 20% (n = 40) and lowest 20% (n = 40) of the BW-ranked ducks as the high and low groups, respectively, and compared BW between these two groups. Not surprisingly, the result showed a significant difference in BW (3.05 vs. 2.49 kg) between the high and the low groups (**Figure 2B**, *P* < 0.0001).

### The Tight Junction Proteins and Cytokines of Muscovy Ducks No Difference Between the High Group and the Low Group

**Figure 3:**
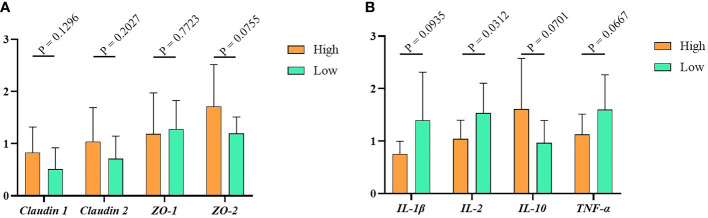
The immunity data of Muscovy Ducks. Two hundred newly hatched ducklings were fed commercial feeds for 70 days before being weighed individually and collected the ileal segment and contents after dissection. The highest 20% and the lowest 20% of the BW-ranked ducks were set as the high and low groups from the 200 Muscovy ducks, respectively. Real-time PCR was used to measure the relative expression of tight junctions **(A)** and cytokines **(B)** in the high and low groups with 40 ducks per group. Data were expressed as mean ± SD (n = 40) and analyzed by unpaired two-tailed Students’ *t*-test.

To detect the relative expression of tight junction proteins and cytokines in the high and low groups, we used RT-PCR to measure the gene expression level of tight junction (*Claudin1*, Claudin2, *ZO-1*, *ZO-2*) and cytokines (*IL-1β*, *IL-2*, *IL- 10*, *TNF-*α) in the ileum tissue. The results indicated that, compared with the low group, the tight junction proteins expression in the high group were higher, but the difference between them was not significant (**Figure 3A**, *P* > 0.05). Similarly, as shown in **Figure 3B**, the relative expression levels of the pro-inflammatory factors *IL-1β*, *IL-2* and *TNF-α* were higher in the low group. In contrast, the expression of anti-inflammatory factor *IL-10* was lower in the high group. Of note, the expression of cytokines other than *IL-2* is not significantly different in the high and low groups.

### Gut Microbiota Differentially Distributed in the High and the Low Groups

**Figure 4:**
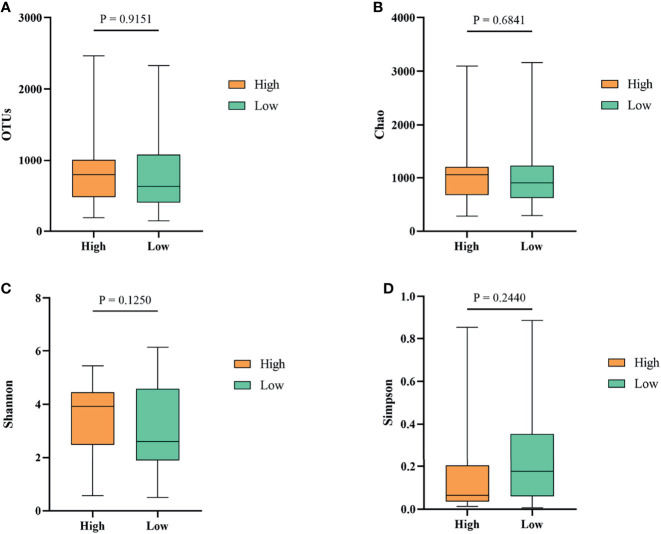
The α-diversity of the ileal microbiota of Muscovy ducks in the high and low groups. The OTUs **(A)**, Chao **(B)**, Shannon **(C)**, and Simpson **(D)** were determined to elucidate the diversity and richness of the microbial community in the ileum of the Muscovy ducks in the high and low groups. Data were expressed as mean ± SD (n = 40) and analyzed by unpaired two-tailed Students’ t-test.

To investigate whether gut microbiota contributed to the BW difference between the high and low groups, we collected ileum content from ducks of high and low groups. After isolating genomic DNA, 16S rRNA sequencing was performed to study the ileal microbiota diversity and structure. As shown in **Figure 4A**, the OTUs obtained from ileal microbiota in ducks of the high group with an average of 728.23 was similar to the reads of ileal microbiota in the low group ducks with an average of 735.01 (*P* > 0.05). The α-diversity was determined by the Chao, Shannon, and Simpson indexes (**Figure 4**). The Chao index, which accounts for the richness of a community, showed that ducks in the high group harbored a more prosperous microbial species than the low group (**Figure 4B**, *P* > 0.05). Nevertheless, considering the evenness, there was no significant difference in Shannon and Simpson indexes of ileal microbiota between the two groups (**Figures 4C, D**, *P* > 0.05).

For ileal microbial composition, the top 6 phyla were Firmicutes, Bacteroidetes, Proteobacteria, Fusobacteria, Campilobacterota, and Planctomycetes in both of the high and low groups, which was coincident with the bacterial composition in the whole population of 200 ducks (**Figure S1**). The relative abundance of the top three phyla, Firmicutes, Bacteroidetes, and Proteobacteria, were similar in the high and low groups, which accounted for 89.05% and 84.77%, respectively. However, Bacteroidetes phyla showed a relatively higher abundance in the high group (28.77%) than in the low group (24.60%). Additionally, ducks in the high group had a relatively lower abundance of Firmicutes, Proteobacteria and Fusobacteria than the low group. At genus level, 7 out of the top 10 abundant genera were shared in the two groups with a pretty different abundance, namely *Candidatus Arthromitus*, *Fusobacterium*, *Vibrio*, *Streptococcus, Clostridium sensu stricto 1*, *Romboutsia*, and *Bergeyella* (**Figure S1**). The other three genera in the high group were *Bacteroides*, *Enterococcus*, and *Lactobacillus*, while *Escherichia-Shigella*, *Terrisporobacter*, *and Turicibacter* were the other 3 of the top 10 genera in the low group.

**Figure 5:**
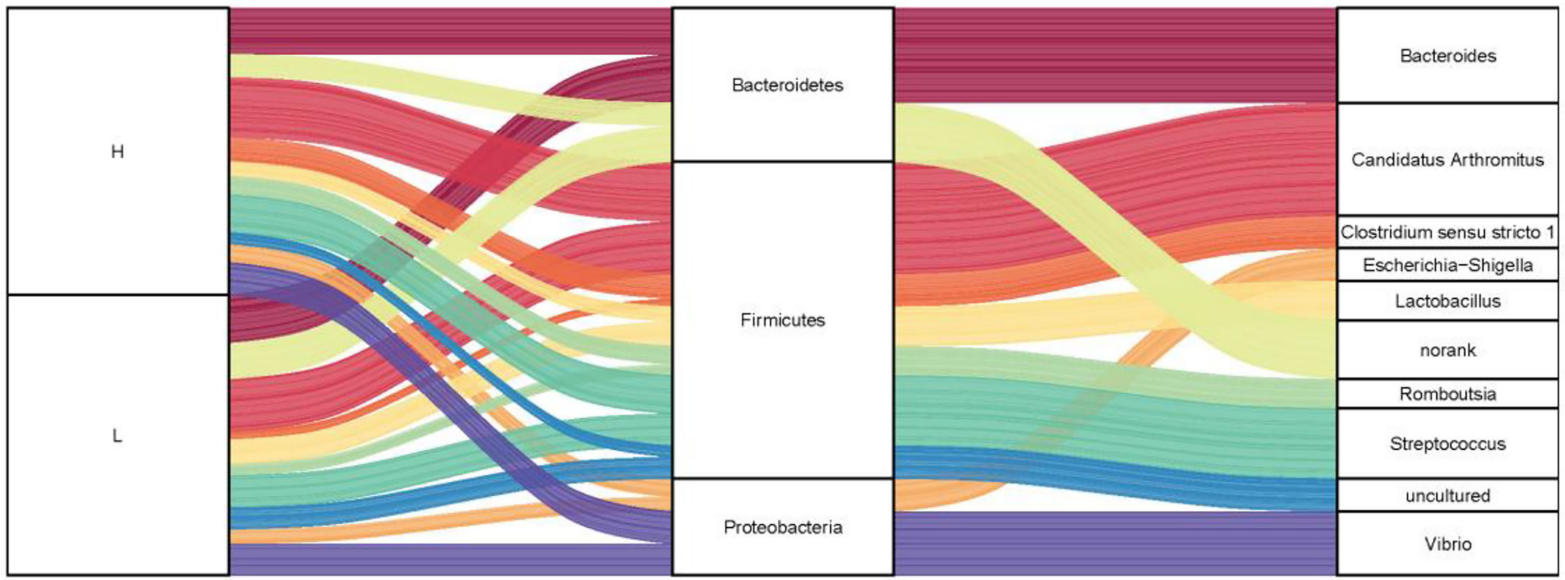
The microbial composition of the ileum of Muscovy ducks in high and low groups. Two hundred newly hatched ducklings were fed commercial feeds for 70 days before the ileal content samples were collected from each ileum segment. The Sankey Diagram (A) was constructed with the relative abundance of OTUs to determine the bacteria composition of the ileum in the high and low groups with 40 ducks per group.

To investigate the microbiota composition in the high and low groups, we calculated the relative abundance of each phylum and genus shown in a Sankey diagram (**Figure 5**). In the figure, the lines of different colors represent different bacterial genera, and the height of the rectangles indicates the relative abundance in the groups (left), phyla (middle), and genera (right). At the phylum level, three predominant phyla were Firmicutes, Bacteroidetes, and Proteobacteria, same with the results of 200 ducks. At the genus level, the top eight genera were *Candidatus Arthromitus*, *Bacteroides*, *Clostridium sensu stricto 1*, *Lactobacillus*, *Streptococcus*, *Romboutsia*, *Vibrio*, and *Escherichia*-*Shigella*. In the top 10 genera, *Bacteroides* and norank were from the phylum Bacteroides, *Candidatus Arthromitus*, *Romboutsia*, *Streptococcus*, *Lactobacillus*, *Clostridium sensu stricto 1* and uncultured from the phylum Firmicutes, *Escherichia*-*Shigella* and *Vibrio* were from the phylum Proteobacteria. Interestingly, the relative abundance of *Bacteroides* and *Candidatus Arthromitus* (16.90% and 19.10%) in the high group of ducks were much higher than the low group (2.76% and 5.57%). In contrast, the relative abundance of *Escherichia*-*Shigella*, *Romboutsia*, *Streptococcus*, *Clostridium sensu stricto 1*, and *Vibrio* in the high group were lower than the low group (1.15% vs. 4.83%, 2.04% vs. 3.06%, 2.69% vs. 12.18%, 2.13% vs. 2.97% and 3.37% vs. 10.33%; **Figure S1**).

### The Relationship Between BW-Related Bacteria and Immunity in the High and Low Groups

**Figure 6:**
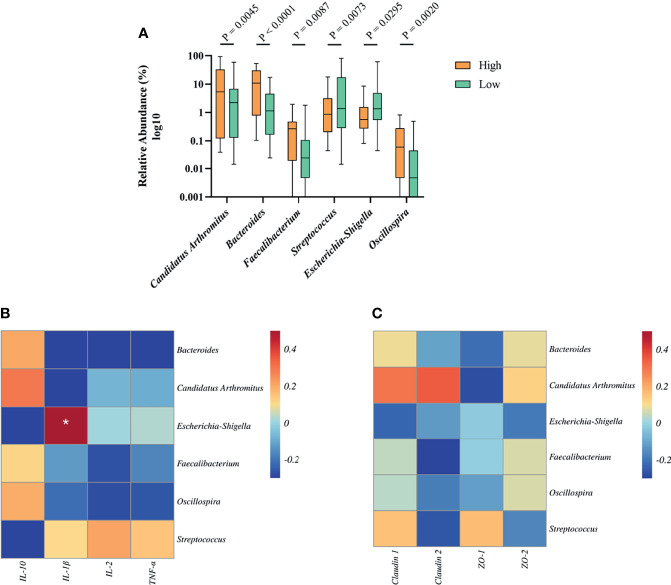
The relationship between BW-related bacteria and immunity of Muscovy ducks in high and low groups. Two hundred newly hatched ducklings were fed commercial feeds for 70 days before the ileal content samples were collected from each ileum segment. **(A)**The indicated OTUs was identified from the genera with significantly different abundance between the high and low groups (n = 40). Data were expressed as mean ± SD (n = 40) and analyzed by unpaired two-tailed Students’ t-test. The correlation heatmap was based on the Pearson algorithm to analyze the correlation between BW-related bacteria and cytokines **(B)** as well as tight junctions **(C)** of Muscovy ducks in high and low groups (n = 40). *P < 0.05.

To determine the relationship between growth-related microbiota and immunity. Above all, we explored the specific bacteria correlated with BW and identified the taxa of which the abundance was significantly different between the high and low groups. The six BW-related genera were *Candidatus Arthromitus*, *Bacteroides*, *Faecalibacterium*, *Streptococcus*, *Escherichia*-*Shigella*, and *Oscillospira* (**Figure 6A**). Among these, we found that the abundance of *streptococcus* (2.69%) and *Escherichia*-*Shigella* (1.15%) in the high group was significantly lower than that in the low group (12.18%, 4.83%), indicating that these two genera were negatively correlated with BW. However, the situation with the bacterial genera *Candidatus Arthromitus*, *Bacteroides*, *Faecalibacterium*, and *Oscillospira* in high and low groups was opposite, which indicated a positive correlation with BW.

Next, we investigated whether the tight junctions and cytokines were associated with BW-related genera. The correlation heatmap was used to show the correlation between them, respectively. As shown in **Figure 6B**, *Escherichia*-*Shigella* was positively correlated with *IL-1β* (*P*<0.05). Other BW-related bacteria were related to cytokines, but there was no significant difference. Similarly, we found that BW-related bacteria are related to tight junctions and tend to differ even though they are not significant (**Figure 6C**, *P*<0.05). This result indicated that the bacteria in the ileum might be related to the intestinal barrier and immunity.

## Discussion

The Muscovy duck, known for its strong adaptability, fertility, fast growth, and high meat yield (32), is native to tropical areas of Central and South America. The composition and functions of intestinal microorganisms play an essential role in the host’s immune homeostasis, especially the intestinal immunity, which will affect the digestion and absorption of nutrients for the host, therefore resulting in the modulation of the host growth (33, 34). This investigation aims to determine whether the bacteria in the ileum might impact the immune status of Muscovy ducks by using 16S rRNA sequencing and correlation analysis among the ileal microbiota, immunity, and growth phenotypes. Through the correlation analysis between BW-related bacteria and immunity, the mechanism of microbes affecting the growth performance of Muscovy ducks was explored.

In the present study, we analyzed the ileal microbiota composition in Muscovy ducks at 70-day old. Firmicutes, Proteobacteria, and Bacteroidetes were the top three predominant phyla in the ileum of Muscovy ducks (**Figure 1**). Accumulated studies have proved that ileal bacteria are primarily composed of the Firmicutes, Proteobacteria and Bacteroidetes in weaned rabbits (35) and weaned piglets (36), while Firmicutes and Proteobacteria are the main phyla in the ileum of yellow broilers (37), suggesting the similarity of microbial composition at the phylum level between Muscovy ducks and other vertebrates. In broiler chickens, the dominant bacteria phyla in the ileum were Firmicutes and Actinobacteria (38). Furthermore, the representative taxa in the three segments of the small intestine in Gaoyou ducks were Firmicutes, Proteobacteria and Bacteroides (39), which complies with our results. Therefore, the difference in ileal microbiota among different animals exists with similarity among different duck species due to different species having different dietary habits.

We hypothesized that the microbes in the ileum might be involved in regulating the growth of Muscovy ducks, leading us to investigate how microbes play an essential role in improving BW. Through correlation analysis in the taxa with significantly different abundances and tight junctions as well as cytokines between the high and low groups, we found that microbes could affect Muscovy ducks’ growth by altering the intestine’s immune status. The immune status of an individual host is defined as the ability of the host to produce an immune response or protect itself from diseases or foreign substances, including tight junction functions, expression of cytokines, and so on (40). Tight junctions, as an important part of the intestinal barrier, can effectively prevent harmful bacteria in the lumen from invading the intestinal epithelium and are an indispensable presence in intestinal immunity (41, 42). The expression level of cytokines can be used to assess the immune status of the intestinal tract, in which pro-inflammatory factors and anti-inflammatory factors are in a dynamic equilibrium state (34). Both tight junctions and cytokines can be adjusted by altering the structure of the intestinal flora. In the present study, we discovered that *Escherichia-Shigella* and *Streptococcus* were negatively correlated with BW, while *Escherichia-Shigella* was positively correlated with *IL-1β* (*P* < 0.05). With IL-1β and IL-10 being markers of inflammatory and pro-inflammatory in the intestine, respectively (43, 44), this result indicates that *Escherichia-Shigella* might decrease the BW of Muscovy ducks by increasing the expression of *IL-1β* and decreasing the *IL-10* expression, which was coincident with the previous study (45). They found that the expression of IL-1β increased in *Escherichia coli* (ETEC) K88-induced diarrhea in weaned piglets, turning into the decreased BW with a decrease in IL-10 expression. Similarly, in a study of the relationship between intestinal bacteria and brain inflammation, it was found that IL-β in patients was positively correlated with *Escherichia-Shigella*, which was defined as inflammatory bacteria (46). Furthermore, *Escherichia-Shigella* was one of the potential pathogens in the intestinal tract. It could induce intestinal inflammation (47) when the intestinal flora is disturbed.

Other newly-identified microbes positively related to body weight were *Candidatus Arthromitus*, *Bacteroides*, *Faecalibacterium*, and *Oscillospira*. *Candidatus Arthromitus* are segmented filamentous bacterium (SFB) belonging to Clostridiaceae. These commensal bacteria could produce around papillary protrusion to colonize the epithelial cells of the vertebrate ileum without causing inflammation. They are believed to be beneficial to the immunity development, maturation of the host’s intestinal tract, and disease prevention (48, 49). *Candidatus Arthromitus* mainly induced naive CD4+ T cells changing into antigen-specific Th17 CD4+ cells (48), which would synthesize IL-17A. Combining with IL-22, IL-17A can promote the expression of antimicrobial peptides and tight junctions to promote the intestinal mucosal barrier (50). Many studies have proved that the immunity induced by *Candidatus Arthromitus* can effectively prevent the colonization of harmful bacteria, such as the intestinal pathogenic *Escherichia coli O103* (51) and *Salmonella* typhimurium (52). However, in the present study, *Candidatus Arthromitus* did not significantly correlate with tight junctions and cytokines. It may limit the number of genes or samples tested in this study.

As a prominent genus within the Bacteroidetes phylum, the obligate anaerobic *Bacteroides* is widely found in the intestines of poultry (24). *Bacteroides* can effectively degrade long-chain polysaccharides, produce short-chain fatty acids (SCFAs) (53)and improve the intestinal environment of beneficial microorganisms (54). SCFAs can be produced by fermentation in most parts of the poultry intestines and positively affect the intestinal barrier, body immunity and promote animal growth (55). However, *Bacteroides* fragilis, as one species of *Bacteroides*, was part of the normal intestinal microbes, but it was considered an important pathogen in clinical practice (56). In the establishment of an intestinal inflammation model using lipopolysaccharide (LPS) and interferon-γ (IFN-γ) as exogenous stimulants of enteric glial cells (EGCs), *Bacteroides fragilis* up-regulated the expression of IL-1β to promote inflammation (57). Moreover, *Bacteroides fragilis* contains polysaccharide A, an immunomodulatory bacterial molecule regulating the immune system by inducing regulatory T cells and producing potent anti-inflammatory IL-10 (58). This was coincident with our results that the relative abundance of Bacteroides was positively correlated with the *IL-10* expression and negatively correlated with the expression of *IL-1β*, *IL-2*, and *TNF-α*. It might be because the pathogenic *Bacteroides* species might contribute more to the detected Bacteroides genus in the present study. Therefore, the specific *Bacteroides* species will be studied in our further investigation.

In recent years, *Faecalibacterium* has attracted much attention as the most promising next-generation probiotics. Currently, the only known species in the genus *Faecalibacterium* is *Faecalibacterium prausnitzii* (*F. prausnitzii*) which is considered a biomarker of intestinal health (59). Newly emerging evidence suggests that *F. prausnitzii* has an important role in maintaining the health of metabolism, SCFAs production and the development of the immune (60). *F*. *prausnitzii* mainly protect the host by producing butyrate and inducing immune cells to produce IL-10, an anti-inflammatory factor (61). In diabetic mice, the active product of F. prausnitzii could repair the intestinal barrier and increase the expression of ZO-1 (62). Consequently, *Faecalibacterium* might be another taxon with the potential to improve the immunity of Muscovy ducks.

*Oscillospira* is a genus of anaerobic bacteria from Clostridial cluster IV and is considered a health-related bacteria (63). Although common, it is rarely cultivated (64). It has been reported that oral administration of *Bifidobacterium longum* strain BR-108 increased the abundance of *Oscillospira* in the ileum, which was positively correlated with the expression of IL-10 (65). In addition, in the study of the perinatal syndrome in sows, it was found that the abundance of *Oscillospira* decreased in the early lactation, which was positively correlated with IL-10 and negatively correlated with TNF-α, but the difference was not significant (66). This was consistent with our results (**Figure 6**). Accumulated evidence suggested that the abundance of *Oscillospira* was reduced when people had inflammatory diseases because *Oscillospira* probably benefited the host by producing butyrate (63, 67), and study has indicated that butyrate was positively correlated with IL-10 and negatively correlated with IL-1β (68). Similarly, another study showed that *Oscillospira* in the intestines of calves before weaning was positively correlated with single nucleotide polymorphisms involved in regulating host immunity (69). These might be other ways *Oscillospira* can enhance the host’s immunity, and the specific mechanism needs further study.

In summary, we identified *Streptococcus* and *Escherichia*-*Shigella* to be negatively related to body weight while *Candidatus Arthromitus*, *Bacteroides*, *Faecalibacterium*, and *Oscillospira* were found to be positively related to body weight of Muscovy ducks by 16S rDNA sequencing. There might be a correlation between BW-related bacteria and tight junctions as well as cytokines. However, this correlation is needed to be further confirmed. These findings could provide basic data for the ileal microbial community of ducks, and their relationship with growth performance, which is conducive to the development of the next generation of probiotics for livestock even for humans and contributes to the duck industry’s development.

## Data Availability Statement

The datasets presented in this study can be found in online repositories. The names of the repository/repositories and accession number(s) can be found below: https://www.ncbi.nlm.nih.gov/, PRJNA762153.

## Ethics Statement

The animal study was reviewed and approved by Animal Care and Use Committee of the Zhejiang Academy of Agricultural Sciences (2019ZAASLA37).

## Author Contributions

Experiment design: HY, CY, WW, and ZF. Animal experiments: ZF, XW, and LL. Data analysis and visualization: ZF, WL, and YX. Roles/writing—original draft: ZF and WL. Writing review and editing: ZF, WL, and YX. All authors contributed to the article and approved the submitted the manuscript.

## Funding

This work was supported by the State Key Laboratory for Managing Biotic and Chemical Threats to the Quality and Safety of Agro-products, Zhejiang Academy of Agricultural Sciences (2010DS700124-ZZ1905), and the China Agriculture Research System of MOF and MARA (CARS-42-27).

## Conflict of Interest

The authors declare that the research was conducted in the absence of any commercial or financial relationships that could be construed as a potential conflict of interest.

## Publisher’s Note

All claims expressed in this article are solely those of the authors and do not necessarily represent those of their affiliated organizations, or those of the publisher, the editors and the reviewers. Any product that may be evaluated in this article, or claim that may be made by its manufacturer, is not guaranteed or endorsed by the publisher.
