# Supplementary material for: Ileal Microbiota Alters the Immunity Statues to Affect Body Weight in Muscovy Ducks
Source: Front Immunol. 2022 Feb 10;13:844102. doi: 10.3389/fimmu.2022.844102 (PMC8866836; doi:10.3389/fimmu.2022.844102)
Supplement: Supplementary file 1 [file Image_1.pdf]

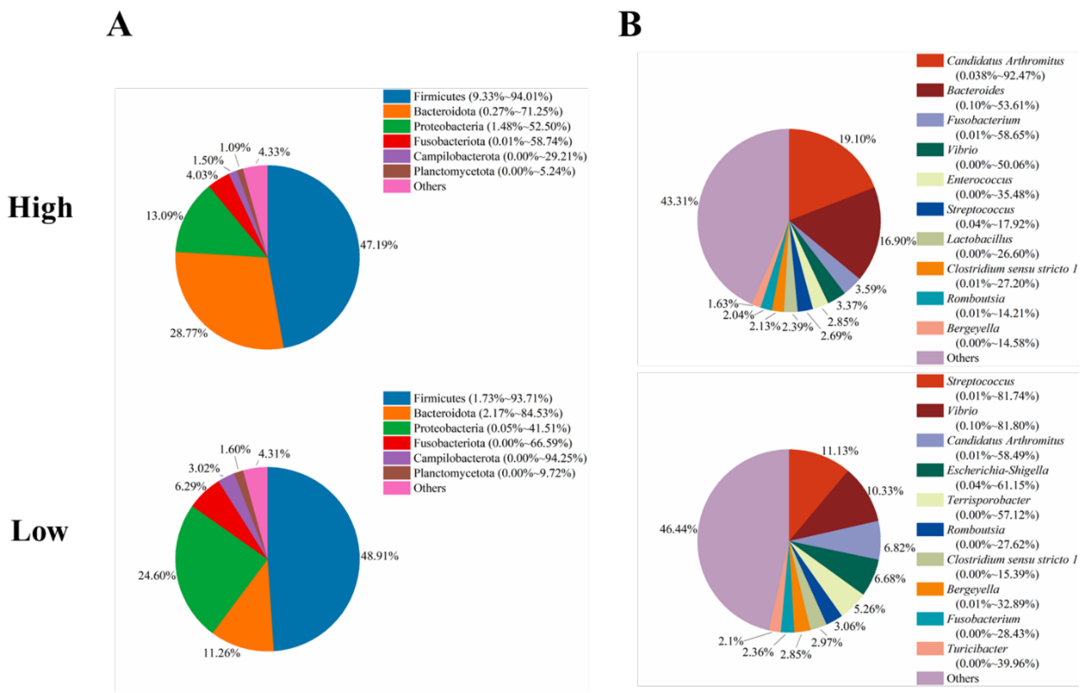

Figure S1. The microbiota composition in the ileum of Muscovy ducks. Two hundred newly hatched ducklings were fed commercial feeds for 70 days before the ileal content samples were collected from each ileum segment. The relative abundance of features was used to determine the composition of ileal bacteria in the high and low groups at the phylum (A) and genus (B) levels. Only the top 6 phyla and 10 genera are shown, with unidentified and lowly abundant bacteria collectively denoted as "Ohers".
